# Does Concentrated Exposure Treatment for Obsessive-Compulsive Disorder Improve Insomnia Symptoms? Results From a Randomized Controlled Trial

**DOI:** 10.3389/fpsyt.2021.625631

**Published:** 2021-08-19

**Authors:** Kristen Hagen, Håkon Nordahl, Gunvor Launes, Gerd Kvale, Lars-Göran Öst, Sigurd Hystad, Bjarne Hansen, Stian Solem

**Affiliations:** ^1^Department of Psychiatry, Molde Hospital, Møre og Romsdal Hospital Trust, Molde, Norway; ^2^Bergen Center for Brain Plasticity, Haukeland University Hospital, Bergen, Norway; ^3^Department of Mental Health, Norwegian University of Science and Technology, Trondheim, Norway; ^4^Department of Clinical Psychology, University of Bergen, Bergen, Norway; ^5^Clinic for B4DT, Haukeland University Hospital, Bergen, Norway; ^6^Sørlandet Sykehus, Department of Psychiatry, Kristiansand, Norway; ^7^Department of Psychology, Stockholm University, Stockholm, Sweden; ^8^Faculty of Psychology, Center for Crisis Psychology, University of Bergen, Bergen, Norway; ^9^Department of Psychology, Norwegian University of Science and Technology, Trondheim, Norway

**Keywords:** obsessive-compulsive disorder, RCT, sleep disturbances, Bergen 4-day treatment, insomnia

## Abstract

Insomnia is a substantial problem in patients with obsessive-compulsive disorder (OCD). There is, however, a lack of studies investigating changes in concurrent symptoms of insomnia in OCD after concentrated treatment. A recent randomized controlled trial randomized participants to the Bergen 4-day treatment (B4DT, *n* = 16), or 12 weeks of unguided self-help (SH, *n* = 16), or waitlist (WL, *n* = 16). Patients from the SH- and WL-group who wanted further treatment after the 12 weeks were then offered the B4DT (total of 42 patients treated with the B4DT). There were no significant differences in symptoms of insomnia between the conditions at post-treatment, but a significant moderate improvement at 3-month follow-up for patients who received the B4DT. Insomnia was not associated with OCD-treatment outcome, and change in symptoms of insomnia was mainly related to changes in depressive symptoms. The main conclusion is that concentrated exposure treatment is effective irrespective of comorbid insomnia, and that insomnia problems are moderately reduced following treatment.

## Introduction

Obsessive-compulsive disorder (OCD) is characterized by recurrent and anxiety provoking thoughts, images and/or impulses, that create the urge to perform compulsive behaviors or mental acts to reduce anxiety (1). Lifetime prevalence is estimated to be 1.6% (2), and without adequate treatment, OCD tends to be chronic with fluctuating intensity (3). Prevalence of comorbid disorders in OCD are reported to be relatively high, and depression and anxiety disorders are most common (1).

Given the extensive suffering many patients with OCD experience, little attention has been devoted to comorbid insomnia problems (4). Insomnia is in general linked to psychopathology, higher symptom severity, and lower levels of emotional functioning and quality of life (5, 6). For OCD there has been found both subjective and objective alterations in sleep patterns (7). Research suggests reduced total sleep time and sleep efficiency in OCD (8). Later bedtime also predicts prospective increase in OCD-symptoms (9). Some studies have found that comorbid sleep disturbance in OCD is accounted for by other factors such as depressive symptoms (10). However, a recent review argues that OCD (but not OC-related disorders) has an unique relation to sleep disturbance that is not better accounted for by negative affect (11), and that its impact on OCD treatment outcome is unclear.

Comorbid sleep disturbances in OCD and anxiety disorders are not automatically resolved after treatment, and only moderate changes in sleep symptomatology can be expected (4). Despite clinical experience indicating that patients with OCD frequently report sleep disturbances (12–14), few studies have investigated whether self-reported insomnia improves after OCD treatment (15). There is therefore a need for more studies that investigate the role of insomnia in OCD treatment outcome among adult samples (7).

Since 2012, the OCD-team in Bergen, Norway, has developed a concentrated exposure-based treatment for OCD, the Bergen 4-day treatment (B4DT), where the treatment is delivered during four consecutive days. In a number of open trials it has been shown that 90% of the patients have responded at post-treatment assessment, and that nearly 70% are in remission at 3-month follow-up (16). Furthermore, the change is maintained at 6 months, 1 year, and 4 years after treatment (17–19).

In a previous effectiveness study (20), we explored whether concurrent self-reported insomnia in patients with OCD improved following the B4DT. There was a significant change in symptoms of insomnia from pre-treatment to 6-month follow-up, however residual symptoms were observed. The study also indicated that insomnia did not impair the primary treatment outcome, and that there was a reduction in insomnia symptoms independent of depressive symptoms. The Nordahl et al. (20) study had an open trial design, which did not allow inferring any causality between the intervention and improvement of the self-reported insomnia. We have now conducted a randomized controlled trial, primarily focusing on the effects of B4DT when compared to a self-help (SH) intervention, and a waiting list (WL) condition with a 6-month follow-up (21). The trial confirmed the results from the previous open trials, and showed that 94% of the patients who had received the B4DT responded post-treatment compared to 13% in the self-help condition, and 0% in the waiting-list condition. At 3- and 6-month follow-up, 69 and 75% of the patients who had received the B4DT were in remission. Also, the trial showed significant changes in comorbid depressive symptoms.

The current study is, to the best of our knowledge, the first controlled trial investigating changes in self-reported insomnia in patients with OCD. The aims of this study were: (1) To investigate differences in self-reported insomnia between patients who received B4DT with patients who received unguided self-help or were on a waitlist, (2) To investigate if insomnia is associated with treatment outcome for the primary OCD-disorder, (3) To investigate the relationship between depressive symptoms and insomnia. Based on our previous open trial (20), as well as the clinical results from the RCT (21), we expected that patients treated with the B4DT would show more improvement in self-reported insomnia than patients in the SH and WL groups. Furthermore, and based on the previous study (20), we hypothesized that symptoms of insomnia would not be associated with treatment outcome. We had no clear hypothesis regarding the relationship between depression and insomnia due to different findings in the research literature.

## Methods

### Design and Procedure

The current study is part of a randomized controlled trial that was carried out at Solvang, Sørlandet Hospital, in Norway, between August 2016 and September 2018. The study protocol was approved by the Regional Committee for Medical and Health Research Ethics of Western Norway, Bergen (REK Vest-2016/794) and was registered in ClinicalTrials.gov (Identifier: NCT02886780). All participants provided written informed consent before inclusion in the study. For a full description of the study procedures, please refer to Launes et al. (21). Eligible patients referred from their general practitioner to the OCD-team, which is part of the public specialist outpatient mental health care, were randomized to either the B4DT (*n* = 16), a 3-month unguided self-help (SH; *n* = 16) based on a manual by Foa and Kozak (22), or a 3-month waiting list (WL; *n* = 16). All patients were assessed the week prior to the intervention, as well as within a week after the intervention ended (in this case, 1 week post-treatment for the B4DT and after 3 months for the SH and WL conditions). The patients randomized to the B4DT were also assessed at 3-month follow-up.

Patients randomized to the SH- or WL-condition who wanted further treatment were offered the B4DT after the initial intervention. Twenty-six of these 32 patients (81.3%) started the B4DT after the initial intervention. Two patients had improved and four patients were not interested in further treatment. Therefore, a total of 42 patients eventually received the B4DT either as initial treatment or after SH or WL [see (23) for further details].

Referred patients were seen for screening and a diagnostic interview to ascertain an OCD diagnosis according to the Diagnostic and Statistical Manual of Mental Disorders (DSM-5). OCD symptom severity was assessed with the Yale-Brown Obsessive Compulsive Scale (24). The screening and interviews were carried out by trained and experienced psychologists at the local OCD-team. Eligible patients were then informed about the study, randomization, and the various conditions.

Inclusion criteria were as follows: the outpatients had to be 18 years or older and fluent in Norwegian, fulfilling diagnostic criteria of OCD according to the DSM-5 and have a Y-BOCS total score of 16 points (indicative of moderate severity) or higher. Exclusion criteria were as follows: ongoing substance abuse/dependence, bipolar disorder or psychosis, ongoing suicidal ideation, intellectual disability (based on previous medical history), and ongoing severe eating disorders. If patients were using antidepressants without having been on stable dosage 4 weeks before the intervention, or if they were unwilling to remain on a stable dosage during the four intervention days, they were excluded from participating. Patients had to be willing to refrain from anxiety reducing substances, such as anxiolytics and alcohol during the 2 days of exposure. Medical records indicated that the included patients complied with these pre-requisites. Patients with a full course of prior CBT for OCD were also excluded, due to an ongoing national trial for treatment non-responders. Patients who fulfilled all inclusion criteria and none of the exclusion criteria were offered participation. After they had signed the informed consent, they were randomized to one of the three conditions.

### Participants

Table 1 displays a summary of the background characteristics of the sample. Mean age for the total sample was 30 years, and the majority of the sample were women (79%). With respect to occupation, 35% reported being employed, 19% were full-time students, and 46% received welfare benefits. A total of 64% were single. Forty-two (87%) of the patients had comorbid disorders (range 1–6). Nine (19%) patients fulfilled the criteria for insomnia disorder. Fourteen patients (29%) had one comorbid disorder, 11 (23%) had two, six (12.5%) had three, seven (14.5%) had four, three (6%) had five, and one (2%) person had six comorbid disorders. There were no significant differences between the conditions in total number of comorbid disorders, but there were significantly more patients with comorbid depression in the SH- and WL- condition, compared to the immediate B4DT-condition.

**Table 1:** Demographic characteristics at pre-treatment.

	**Total (*N* = 48)**	**B4DT (*n* = 16)**	**SH (*n* = 16)**	**WL (*n* = 16)**	***χ^2^/F***	***p***
**Demographics**	***M*** **(** ***SD*** **)**					
Age	30.35 (11.08)	33.35 (14.93)	27.75 (7.09)	30.06 (9.75)	0.99	.38
OCD duration	14.48 (10.15)	13.63 (8.62)	12.88 (10.15)	16.94 (11.64)	0.72	.49
***n*** **(%)**						
Female gender	(79) 38	(88) 14	(81) 13	(69)11	1.77	.41
Single	(44) 21	(38) 5	(63) 10	(38) 6	3.56	.17
Work situation					0.88	.93
Student	(19) 9	(25) 4	(19) 3	(13) 2		
Employed	(35) 17	(31) 5	(38) 6	(38) 6		
Welfare benefits	(46) 22	(44) 7	(44) 7	(50) 8		
Psychotropic drugs	(44) 21	(31) 5	(50) 8	(50) 8	1.52	.47

*B4DT, Bergen 4-day treatment; SH, self-help; WL, waitlist; BIS, Bergen Insomnia Scale*.

Medication use was registered at the initial screening interview; 12 (25%) used SRI/SSRI/SNRI, three (6%) benzodiazepines prescribed for sleep, three (6%) anti-psychotics, two (4%) hypnotics, two (4%) stimulants, and three (6%) used antiepileptic medication. There were no differences between the conditions on the background variables mentioned above.

### The Bergen 4-Day Treatment

The B4DT is a concentrated exposure based OCD treatment delivered during four consecutive days (25) in groups of 3-6 patients with the same number of therapists. The first day of the B4DT consists of a 1.5 h long psychoeducation focusing on the rationale and principles of the B4DT-treatment, and planning of exposure tasks for the next 2 days. Day two and three are dedicated to therapist-assisted exposure focusing on as many OCD-relevant settings as possible. At the end of day three, relatives and friends are invited to a 1 h long psychoeducation about OCD and how they can help. Day four is concentrated on summarizing the previous days and the things the patients have learnt, and planning for how to maintain and integrate the changes in their everyday living the next 3 weeks to come. The B4DT-format does not include any form of specific sleep interventions or information regarding sleep.

### Therapist Competence

Each treatment group conducted in this study was led by a trained expert in the B4DT-format. Prior to becoming an expert the therapists have to have acted as a leader in at least two B4DT-groups and been rated as competent by two B4DT-experts. All therapists participating in this study were trained OCD-therapists and their competence had been evaluated independently by two B4DT-experts. It is also required that the therapists have participated in two or more B4DT-groups under supervision and been rated competent on a modified version of the OCD CORE competencies instrument (26). Before becoming a B4DT-therapist/expert they had to pass a multiple choice exam on the format prior to the start of the present study.

### The Self-Help Intervention

The self-help book used was written by Foa and Kozak (22), and covers psychoeducation about OCD, and gives the reader a basic understanding of the use of exposure and ritual prevention (examples on how to perform exposure tasks are presented for the reader). The book does not include any information regarding sleep or specific sleep interventions. The self-help condition lasted 12 weeks from pre- to post assessment. The patients had no contact with the therapists during the treatment period (unguided self-help).

### Assessment

*Yale-Brown Obsessive-Compulsive Scale* (24, 27); is regarded as the gold standard clinical interview when assessing severity of obsessive-compulsive symptoms (28). The interview consists of 10 items, where five items measure obsessions on a Likert scale from 0 (no symptoms) to 4 (severe symptoms), and five items measure compulsions on the same Likert scale. The total score range from 0 to 40, and higher scores indicate more severe OCD-symptoms. The Y-BOCS has demonstrated good psychometric properties (24, 27).

*Bergen Insomnia Scale* [BIS; (29)] is a self-report questionnaire used to assess insomnia symptoms occurring the past month. The questionnaire consists of 6 items, where each item is rated on a Likert scale from 0 (none of the days during the week) to 7 (all of the days during the week). The first three items concern problems with sleep onset, maintenance, and early morning wakening. The last three items refer to not feeling adequately rested, experiencing daytime impairment, and feeling dissatisfied with sleep. Total score range from 0 to 42, with higher scores indicating more severe insomnia. Insomnia is indicated if one or more of the items 1–4 is rated 3 or higher, combined with one of items 5–6 is rated 3 or higher. The construction of BIS was based on the criteria for insomnia in the DSM-IV-TR (30). The BIS has shown to be a reliable and valid instrument for insomnia and has good internal consistency (29). Post-treatment assessment was conducted after completing the 4 day treatment for the B4DT condition, and after 3 months for the SH and WL conditions.

*Patient Health Questionnaire-9* [PHQ-9; (31)] is a self-report questionnaire based on nine criteria for diagnosing depression in DSM-IV. The nine items are rated on a Likert-scale from 0 (not at all) to 3 (nearly every day), and measures the past 2 weeks. A total score from 0 to 27 is achievable, with higher scores reflecting greater depressive severity; mild (5–9), moderate (10–14), moderately severe (15–19) and severe (≥20). The PHQ-9 is found to have good psychometric properties (31).

### Statistical Analyses

The international consensus criteria was adapted for assessing treatment response (32). Treatment response involved a achieving at least a 35% reduction in Y-BOCS score. Remission was defined as meeting the treatment response criterion and having a post-treatment or follow-up Y-BOCS score of 12 or less. Effect sizes were calculated using pooled standard deviations.

In order to test aim 1, hierarchical linear models (HLM) were conducted to investigate differences in self-reported insomnia between treatment conditions. Interactions between time (i.e., measurement points) and treatment condition (B4DT, SH, and WL) were used to identify differences between conditions.

In order to test aim 2 (whether insomnia impacted treatment outcome), changes in Y-BOCS were compared over time (pre-treatment, post-treatment and follow-up) for patients with and without indication of insomnia at pre-treatment. In this HLM, time and group (patients with vs. patients without insomnia) were included as separate fixed effects, as well as a time × group interaction. We also conducted *t*-tests comparing scores on Y-BOCS, PHQ-9, and BIS between patients with and without indications of insomnia at pre-treatment, post-treatment, and follow-up.

To test aim 3 (the relationship between depressive symptoms and insomnia), we conducted a separate HLM with depressive symptoms as a time-varying covariate. To disentangle the within and between effects of depressive symptoms, we first created one variable that represents the between-person effect by subtracting the sample PHQ-9 mean from each patient's overall mean on the three measurement points. Then we created another variable that represents the within-person effect by subtracting the patient mean from each individual's PHQ-9 score.

Following the principle of intention to treat, all participants were included in the analyses, irrespective of missing data at any of the measurement points. For the treatment condition comparison analyses this gave a total *N* of 48, while the total *N* for the effects of the B4DT on insomnia was 42. Hierarchical linear models do not assume balanced data and are able to account for missing data on the response variable by using all available data on each participant, and under the missing at random (MAR) assumption, provide unbiased estimates (33, 34). There was a total of 3% missing data in the dataset.

## Results

### Preliminary Analyses; Change in OCD-Symptoms

At post-treatment the treatment response rate was 93.8% in the immediate B4DT condition compared to 12.5% in SH, and 0% in WL (21). The rates of remission were 62.5, 6.3, and 0%, respectively. For the 26 patients who were treated with the B4DT after initial self-help or waiting list, 59.5% were in remission at post-treatment, 31.0% had treatment response, and 9.5% showed no change (23). At 3-month follow-up for these 26 patients, 71.4% were in remission, 19.0% had treatment response, and 9.5% showed no change. There were no significant differences on Y-BOCS scores between the patients who received the B4DT after first being on the WL- or SH condition (*n* = 26) and those that received the B4DT immediately (*n* = 16), at post-treatment [Y-BOCS = 11.80 (3.16), *F*_(1, 40)_ = 0.61, *p* = 0.441] or at 3-month follow-up [Y-BOCS = 10.54 (5.87), *F*_(1, 40)_ = 1.139, *p* = 0.292].

### Aim 1, Were There Differences in Insomnia Between Conditions?

Among all participants in the RCT, there were 39 (81.3%) with indications of insomnia at pre-treatment. Of the 42 patients who were eventually treated with the B4DT, there were 35 (83.3%) patients with indications of insomnia at pre-treatment. For the three treatment conditions, there were three patients in each arm diagnosed with a sleep disorder. A summary of insomnia symptoms in the three conditions is displayed in Table 2.

**Table 2:** Comparisons of insomnia symptoms in the three treatment conditions.

	**Total (*N* = 48)**	**B4DT (*n* = 16)**	**SH (*n* = 16)**	**WL (*n* = 16)**	***χ^2^/F***	***p***
***N*** **(%)**					
Sleep disorder SCID pre	9 (18.8)	3 (18.8)	3 (18.8)	3 (18.8)	0.00	1.00
Insomnia ind. pre	39 (81.3)	12 (75.0)	14 (87.5)	13 (81.3)	0.82	.66
Insomnia ind. f-u	31 (66.0)	8 (50.0)	12 (75.0)	11 (73.3)	2.61	.30
***M*** **(** ***SD*** **)**					
BIS pre-treatment	17.75 (8.67)	15.00 (7.94)	19.63 (8.25)	18.63 (9.58)	1.28	.29
BIS post-treatment	18.57 (9.48)	15.50 (10.01)	19.94 (8.08)	20.40 (10.08)	1.30	.28
BIS 3-month follow-up	–	11.44 (8.79)	–	–		

*B4DT, Bergen 4-day treatment; SH, self-help; WL, waitlist; BIS, Bergen Insomnia Scale*.

**Figure 1:**
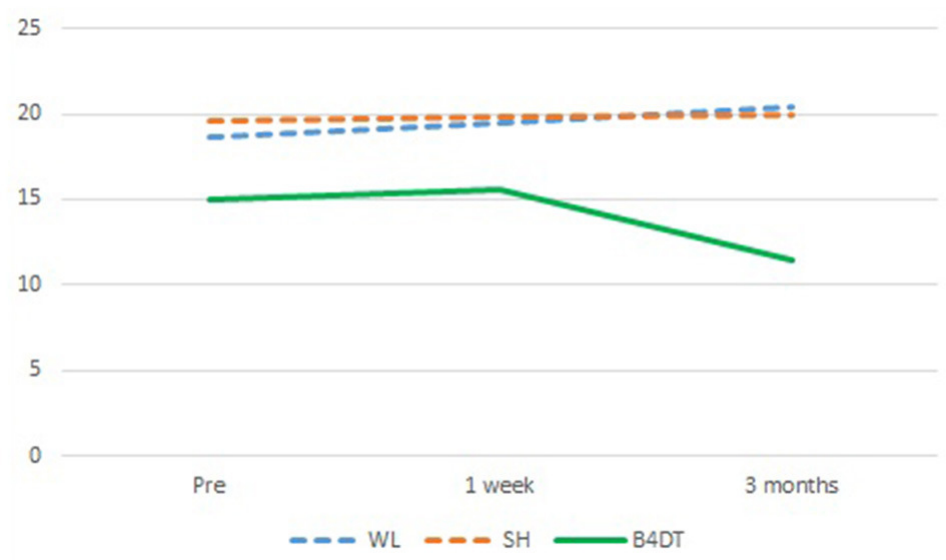
Insomnia scores for the three treatment conditions (*n* = 48) from pre-treatment to 3 months after starting intervention. The B4DT group had post-assessment 1-week after the treatment ended and follow-up 3 months after the intervention started. The WL and SH condition had post-assessment 3 months after the intervention started, which corresponds to the 3-month FU for the B4DT group.

An HLM with time and treatment condition as independent variables revealed no statistically significant effects of time (χ^2^(1) = 0.69, *p* = 0.406) or treatment condition (χ^2^(2) = 3.01, *p* = 0.222). Adding an interaction between time and condition resulted in an overall non-significant interaction effect, χ^2^(2) = 0.32, *p* = 0.852. Thus, there is no evidence that self-reported insomnia differed between patients who received B4DT and patients who received unguided self-help or were on a waitlist. There was a moderate reduction in BIS scores at follow-up for the immediate B4DT group (*d* = 0.43). See Figure 1 for comparison between the groups across time.

To investigate change in self-reported insomnia for all patients who eventually received the B4DT (*n* = 42), a separate HLM was conducted on the subsample who had indication of insomnia pre-treatment according to BIS (*n* = 35; 83.3%). This subsample included patients who had received B4DT either as first treatment (*n* = 16) or after first participating in SH (*n* = 13) or WL (*n* = 13). The analysis showed a statistically significant overall effect of time, χ^2^(2) = 26.22, *p* < 0.001, with a significant decrease in BIS scores from pre-treatment to 3-month follow-up, *b* = −4.79, 95% CI = −7.35, −2.24, *p* < 0.001, but not from pre-treatment to post-treatment, *b* = 0.54, 95% CI = −1.96, 3.04, *p* = 1.00.

### Aim 2, Is Insomnia Associated With OCD Treatment Outcome?

A comparison between the insomnia and non-insomnia groups is presented in Table 3. To test whether insomnia symptoms impaired treatment outcome for B4DT, a separate HLM was conducted to compare Y-BOCS scores over time for patients with and without indication of insomnia at pre-treatment. This analysis included patients who had received B4DT either as first treatment (*n* = 16) or after first participating in SH (*n* = 13) or WL (*n* = 13). The results revealed an overall statistically significant effect of time, χ^2^(2) = 504.21, *p* < 0.001. The patients scored significantly lower at both post-treatment (*b* = −15.58, 95% CI = −17.54, −14.13, *p* < 0.001) and follow-up (*b* = −17.87, 95% CI = −19.58, −16.16, *p* < 0.001) compared with pre-treatment. Including an interaction between time and insomnia (patients with vs. patients without indications of insomnia at pre-treatment) resulted in a statistically significant interaction effect, χ^2^(2) = 6.27, *p* = 0.044. The results showed that patients with insomnia at pre-treatment had a steeper decrease from pre-treatment to follow-up than patients without insomnia, *b* = −5.64, χ^2^(2) = 6.25, *p* = 0.044. There was no statistically significant difference from pre-treatment to post-treatment between the insomnia and non-insomnia groups, χ^2^(2) = 1.89, *p* = 0.169.

**Table 3:** Sample comparison between patients with insomnia indication (*n* = 35) and patients without insomnia indication (*n* = 7).

	**Insomnia**		**No insomnia**			
	***M***	***SD***	***M***	***SD***	***t*/χ^**2**^**	***p***
Age	28.83	8.3	36.14	18.58	1.02	0.344
Women (%)	71.4		77.1		0.102	1.00
**OCD symptoms**						
Y-BOCS Pre	27.80	3.93	26.57	3.60	0.76	0.450
Y-BOCS Post	11.45	3.51	13.33	5.88	−1.15	0.442
Y-BOCS F-U	8.99	5.83	13.40	5.97	−1.82	0.077
**Depression symptoms**						
PHQ-9 Pre	13.63	5.41	8.86	3.76	2.22	0.032^*^
PHQ-9 Post	8.36	3.86	5.86	3.39	1.60	0.119
PHQ-9 F-U	7.73	4.92	6.86	4.06	0.44	0.662
**Insomnia symptoms**						
BIS Pre	20.77	6.84	5.43	1.81	11.42	<0.001^***^
BIS Post	19.23	8.43	7.57	7.01	3.38	0.002^**^
BIS F-U	16.13	7.72	3.57	1.81	8.52	<0.001^***^

*BIS, Bergen Insomnia Scale; Y-BOCS, Yale-Brown Obsessive-Compulsive Scale; PHQ-9, Patient Health Questionnaire 9*.

* *p ≤ 0.05;*

** *p ≤ 0.01;*

*** *p ≤ 0.001*.

This analysis was repeated using continuous BIS scores instead of a dichotomous (insomnia vs. non-insomnia) variable. Including an interaction between time and BIS resulted in a statistically non-significant interaction effect, χ^2^(2) = 2.90, *p* = 0.235. The results showed that pre-treatment BIS did not have a statistically significant effect on Y-BOCS scores at post-treatment (*b* = −0.08, 95% CI = −0.28, 0.12, *p* = 0.451) or follow-up (*b* = −0.17, 95% CI = −0.37, 0.03, *p* = 0.089).

### Aim 3, the Relationship Between Depressive Symptoms and Insomnia

There were significant changes in depression following treatment. This change (pre-treatment to follow-up) was more evident for the insomnia group (*d* = 1.14) than the non-insomnia group (*d* = 0.51). A HLM revealed that both the within-person variation and the between-person variation in depressive symptoms had a statistically significant association with BIS scores. Patients with above average depressive symptoms on average also scored higher on BIS across the three measurement points (*b* = 0.93, 95% CI = 0.39, 1.47, *p* = 0.001). Additionally, those patients who at one time-point exhibited an increase in BIS scores (i.e., who scored above their own average on PHQ) also tended to report an increase in BIS scores (*b* = 0.78, 95% CI = 0.54, 1.03, *p* < 0.001).

Correlation analyses showed that the association between change in BIS and change in PHQ-9 (from pre-treatment to follow-up) was *r* = 0.61. In contrast, the correlation between change in BIS and change in Y-BOCS was *r* = 0.28 and not significant, and *r* = 0.35 for change in PHQ-9 and Y-BOCS.

## Discussion

To the best of our knowledge, this is the first study to investigate changes in self-reported symptoms of insomnia in patients with OCD undergoing treatment in a randomized controlled trial. This topic is clinically relevant as previous studies have shown that symptoms of insomnia are present in 50–80% of all patients with a psychiatric disorder (35, 36). At pre-treatment, 81.3% reported indication of insomnia according to BIS cut-off scores. This reflects a high rate of insomnia in the current group of patients and further add to the growing body of research indicating insomnia as a problem area for people with OCD (7, 37, 38). This finding is also in accordance with our previous finding of 70% insomnia in OCD (12, 13, 20).

Although 81% had indication of insomnia, only 19% met DSM-5 criteria for a sleep disorder. This discrepancy is likely due to differences between DSM-IV and DSM-5 criteria and differences when using self-reported symptoms and diagnostic assessors. The BIS was based on DSM-IV-TR where symptoms only had to last 1 month. In comparison, the DSM-5 criterion is 3 months. Furthermore, indications of insomnia is not the same as diagnosis, but the BIS can be used to screen for insomnia.

There were no statistically significant differences in BIS scores between the three conditions at pre- or post-treatment, but patients in the B4DT condition showed improved sleep at follow-up. This suggests that even though the B4DT is effective in reducing primary OCD symptoms and secondary symptoms (e.g., depression) at post-treatment, symptoms of insomnia did not show a similar immediate improvement. The lack of significant findings post-treatment is most likely due to the brief treatment format and the fact that the BIS measure assesses sleep problems occurring during the past month. Since the B4DT only lasts for 4 days, it would be unrealistic to expect significant differences post-treatment. Another possible explanation might be that symptoms of insomnia alleviate over longer periods of time (than 4 days) as the patients are continuously working with the exposure principles and treatment plan (37, 39). While there was a significant improvement in symptoms of depression scores at post-treatment, a decrease in insomnia symptoms might be more difficult to achieve and detect during this short period of time.

The patients who received B4DT had a significant improvement in self-reported insomnia from pre-treatment to 3-month follow-up (moderate effect size, *d* = 0.53). A possible explanation is that the B4DT has a generic effect as the B4DT is not only effective in treating the principal symptoms, but also has a positive effect on comorbid symptoms. This finding is in accordance with a meta-analysis by Belleville et al. (4) who suggested that CBT-based treatment for anxiety disorders seems to have a moderate effect on concomitant insomnia. Patients who undergo the concentrated ERP treatment learn specific principles to manage thoughts and emotions, and these principles might generalize to how the patients deal with OCD-symptoms (e.g., intrusive thoughts) in the pre-sleep period or during awakenings throughout the night (4).

Furthermore, the results suggest that symptoms of insomnia was not associated with the primary treatment outcome. This is in accordance with our previous effectiveness studies on B4DT and our previous study investigating symptoms of insomnia in treatment seeking OCD-patients (20). This suggests that insomnia should not be considered an exclusion criterion for patients seeking intensive treatment for OCD. Furthermore, the results suggested that patients with insomnia also had higher scores on measures of depression. This is in line with previous research suggesting a close interplay between such symptoms. Results from this trial also dovetails recent suggestions that clinical trials tend to have larger effect on the primary outcome measure (e.g., OCD symptoms) than secondary outcome measures (e.g., quality of life, depression, worry, and insomnia), and that further interventions may be needed to address these (40). Disorder specific treatments may therefore expect residual insomnia and should consider implementing sleep management strategies to improve symptoms of insomnia.

When we statistically controlled for depressive symptoms, the reduction in BIS scores was no longer significant. This may indicate that the improvement in sleep is more related to the reduction in depressive symptoms than to the OCD symptoms itself, as has been reported by other studies (10, 41, 42). However, this is in contrast to our previous study, which indicated that symptoms of insomnia are reduced after treatment, independent of depressive symptoms (20). There is therefore a need for further studies to investigate the role comorbid depression has related to sleep difficulties in OCD.

There are limitations to this study that need to be considered. BIS does not differentiate between insomnia symptoms found in primary insomnia and those found in other sleep disturbances, mental disorders, or due to medical conditions (29). Self-reported measures of sleep can present a challenge as the patients are asked to rate symptoms of their insomnia, and not the processes that creates these symptoms (e.g., obsessions, worry), and therefore it is difficult to determine the source of the insomnia symptoms (that, in fact, could be explained by other factors than the OCD itself), hence the high prevalence rates in this sample. It is recommended that future studies include some form of objective sleep measurements (e.g., actigraphy- and/or polysomnography measurement), clinical interviews (pre- and post-treatment), and sleep diaries to get more accurate information regarding night-to-night variability and function. A replication of the study with different conditions, and with larger sample sizes is warranted to further our understanding of the treatment effect of concentrated ERP treatment on OCD and concurrent symptoms of insomnia.

It should also be noted that the sample consisted of only patients that had not previously received CBT-treatment. The proportion of women (79%) was also particularly high in the current sample. Future studies should consider to investigate long-term follow-up outcome to further explore residual symptoms of insomnia in OCD and how this might affect treatment response and symptom recurrence (42). This could shed light on the possible importance of specific sleep interventions in the treatment of OCD.

In conclusion, the results indicate that symptoms of insomnia in OCD-patients do not impair the primary treatment outcome, and is moderately improved after specific treatment. However, residual insomnia symptoms should be expected and conceivably be dealt with to prevent relapse and recurrence in a follow-up period.

## Data Availability Statement

The datasets generated for this study are available on request to the corresponding author. Requests to access these datasets should be directed to kristen.hagen@helse-mr.no.

## Ethics Statement

The trial received ethical approval from the Regional Committee for Research Ethics (REK Vest, 2016/794) and was registered in ClinicalTrials.gov (Identifier: NCT02886780). The patients/participants provided their written informed consent to participate in this study.

## Author Contributions

GK, L-GÖ, BH, KH, and GL contributed to the study design. GL and KH contributed to the data collection. KH, HN and SS drafted the manuscript. KH, SS and SH conducted the statistical analysis. All authors reviewed and approved the manuscript.

## Conflict of Interest

The authors declare that the research was conducted in the absence of any commercial or financial relationships that could be construed as a potential conflict of interest.

## Publisher's Note

All claims expressed in this article are solely those of the authors and do not necessarily represent those of their affiliated organizations, or those of the publisher, the editors and the reviewers. Any product that may be evaluated in this article, or claim that may be made by its manufacturer, is not guaranteed or endorsed by the publisher.
